# Multimodal integration of blood RNA and ctDNA reflects response to immunotherapy in metastatic urothelial cancer

**DOI:** 10.1172/jci.insight.186062

**Published:** 2025-01-30

**Authors:** Sandra van Wilpe, Davide Croci, Sara S. Fonseca Costa, Iris B.A.W. te Paske, Sofie H. Tolmeijer, Jolique van Ipenburg, Leonie I. Kroeze, Simona Pavan, Sylvain Monnier-Benoit, Guido Coccia, Noushin Hadadi, Irma M. Oving, Tineke J. Smilde, Theo van Voorthuizen, Marieke Berends, Mira D. Franken, Marjolijn J.L. Ligtenberg, Sahar Hosseinian Ehrensberger, Laura Ciarloni, Pedro Romero, Niven Mehra

**Affiliations:** 1 Medical Oncology Department, Research Institute for Medical Innovation, Radboud University Medical Center, Nijmegen, Netherlands.; 2 Novigenix SA, Epalinges, Switzerland.; 3 Department of Pathology, Research Institute for Medical Innovation, Radboud University Medical Center, Nijmegen, Netherlands.; 4 Department of Medical Oncology, Ziekenhuisgroep Twente, Almelo, Netherlands.; 5 Department of Medical Oncology, Jeroen Bosch Ziekenhuis, ‘s-Hertogenbosch, Netherlands.; 6 Department of Medical Oncology, Rijnstate, Arnhem, Netherlands.; 7 Department of Medical Oncology, Canisius Wilhelmina Ziekenhuis, Nijmegen, Netherlands.; 8 Department of Human Genetics, Research Institute for Medical Innovation, Radboud University Medical Center, Nijmegen, Netherlands.

**Keywords:** Immunology, Oncology, Bioinformatics, Cancer immunotherapy, Urology

## Abstract

BACKGROUND. Previously, we demonstrated that changes in circulating tumor DNA (ctDNA) are promising biomarkers for early response prediction (ERP) to immune checkpoint inhibitors (ICIs) in metastatic urothelial cancer (mUC). In this study, we investigated the value of whole-blood immunotranscriptomics for ERP-ICI and integrated both biomarkers into a multimodal model to boost accuracy.

METHODS. Blood samples of 93 patients were collected at baseline and after 2–6 weeks of ICI for ctDNA (*n* = 88) and immunotranscriptome (*n* = 79) analyses. ctDNA changes were dichotomized into increase or no increase, the latter including patients with undetectable ctDNA. For RNA model development, the cohort was split into discovery (*n* = 29), test (*n* = 29), and validation sets (*n* = 21). Finally, RNA- and ctDNA-based predictions were integrated in a multimodal model. Clinical benefit (CB) was defined as progression-free survival beyond 6 months.

RESULTS. Sensitivity (SN) and specificity (SP) of ctDNA increase for predicting non-CB (N-CB) was 59% and 92%, respectively. Immunotranscriptome analysis revealed upregulation of T cell activation, proliferation, and interferon signaling during treatment in the CB group, in contrast with N-CB patients. Based on these differences, a 10-gene RNA model was generated, reaching an SN and SP of 73% and 79%, respectively, in the test and 67% and 67% in the validation set for predicting N-CB. Multimodal model integration led to superior performance, with an SN and SP of 79% and 100%, respectively, in the validation cohort.

CONCLUSION. The combination of whole-blood immunotranscriptome and ctDNA in a multimodal model showed promise for ERP-ICI in mUC and accurately identified patients with N-CB.

FUNDING. Eurostars grant E! 114908 - PRECISE, Paul Speth Foundation (Bullseye project).

## Abstract 2

We predict clinical benefit to immunotherapy by analyzing combined biomarkers from circulating tumor DNA and immune cell gene expression data in blood.

## Introduction

In the last decade, immune checkpoint inhibitors (ICIs) targeting programmed cell death 1 (PD-1) or its major ligand (PD-L1) have become one of the main treatment modalities for patients with irresectable or metastatic urothelial cancer (mUC). In 2017, pembrolizumab became the standard-of-care treatment for patients with mUC following progression on first-line platinum-based chemotherapy based on results of the KEYNOTE-045 trial (1). Since then, the use of ICIs in patients with mUC has shifted to the first-line and maintenance setting. In 2021, maintenance therapy with avelumab became available for patients with a response or stable disease to first-line platinum-based chemotherapy (2). Very recently, the combination of pembrolizumab and antibody-drug conjugate enfortumab-vedotin (EV) became the new standard-of-care first-line treatment based on results of the EV-302 trial. In this phase III clinical trial, pembrolizumab-EV prolonged median overall survival (mOS) from 16.1 to 31.5 months compared with platinum-based chemotherapy in the first-line setting (3, 4). Another new first-line treatment option for patients who are eligible for cisplatin is the combination of nivolumab with cisplatin and gemcitabine, which has shown an OS advantage compared with cisplatin-based chemotherapy alone in the CheckMate 901 trial (5).

Although ICI-containing combination therapies have proven their superiority compared with first-line chemotherapy in unselected fit patients (3–5), it is anticipated that ICI monotherapy will continue to be an important treatment modality. First, ICI monotherapy will continue to play an important role in the treatment of frail or elderly patients with mUC because of the high toxicity associated with combination therapies. In addition, there might be a role for ICI monotherapy in biomarker-selected patients who are predicted to durably benefit from ICI monotherapy, regardless of frailty, to avert unnecessary toxicity and costs.

Responses to ICIs in mUC are heterogeneous. Specifically, ICI monotherapy induces objective response in 20%–25% of mUC patients receiving first- or second-line ICIs and approximately 10% are still progression-free after 4 years (6, 7). These latter patients might not derive extra benefit from the addition of EV or chemotherapy. To personalize treatment decisions in mUC, there is a need for high-precision biomarkers that can identify patients who benefit from ICI monotherapy. Several baseline tumor biomarkers, including tumor mutational burden (TMB), PD-L1 expression, and tumor immune cell infiltration, have been associated with response to ICIs in mUC (8–12). Although PD-L1 expression enriches for responders to first-line ICIs in cisplatin-ineligible patients and is used to select patients for ICIs over carboplatin-based chemotherapy, none of these standalone biomarkers are accurate enough to predict response to ICIs.

In recent years, circulating tumor DNA (ctDNA) measurement has emerged as a method to monitor treatment response (13–15). The ctDNA level correlates well with tumor burden and can, therefore, be used as a noninvasive tool to monitor treatment response. We previously demonstrated that increases in ctDNA after 3–6 weeks are a promising biomarker for the early identification of disease progression to ICIs in mUC (15). However, ctDNA does not capture all host-related and tumor microenvironment–related factors that play a role in antitumor immunity. Multimodal biomarkers capturing both tumor and immune signals might improve biomarker accuracy.

In this study, we searched for on-treatment (OT) biomarkers that accurately identify patients without clinical benefit (N-CB), so that those with N-CB can be considered for other, more effective (combinatorial) therapies, while unwanted treatment discontinuation in patients with clinical benefit (CB) is avoided. We analyzed ctDNA and the peripheral blood immunotranscriptome in baseline (BL) and early OT samples of mUC patients treated with ICI monotherapy. We show that early changes in the peripheral blood immunotranscriptome are associated with response to ICIs and can be utilized to predict CB. Additionally, we demonstrate the synergy between ctDNA and whole-blood RNA-sequencing (RNA-seq) data, by combining the 2 approaches in a multimodal model for early response prediction.

## Results

### Clinical characteristics of the ICI-treated mUC patient cohort.

**Figure 1:**
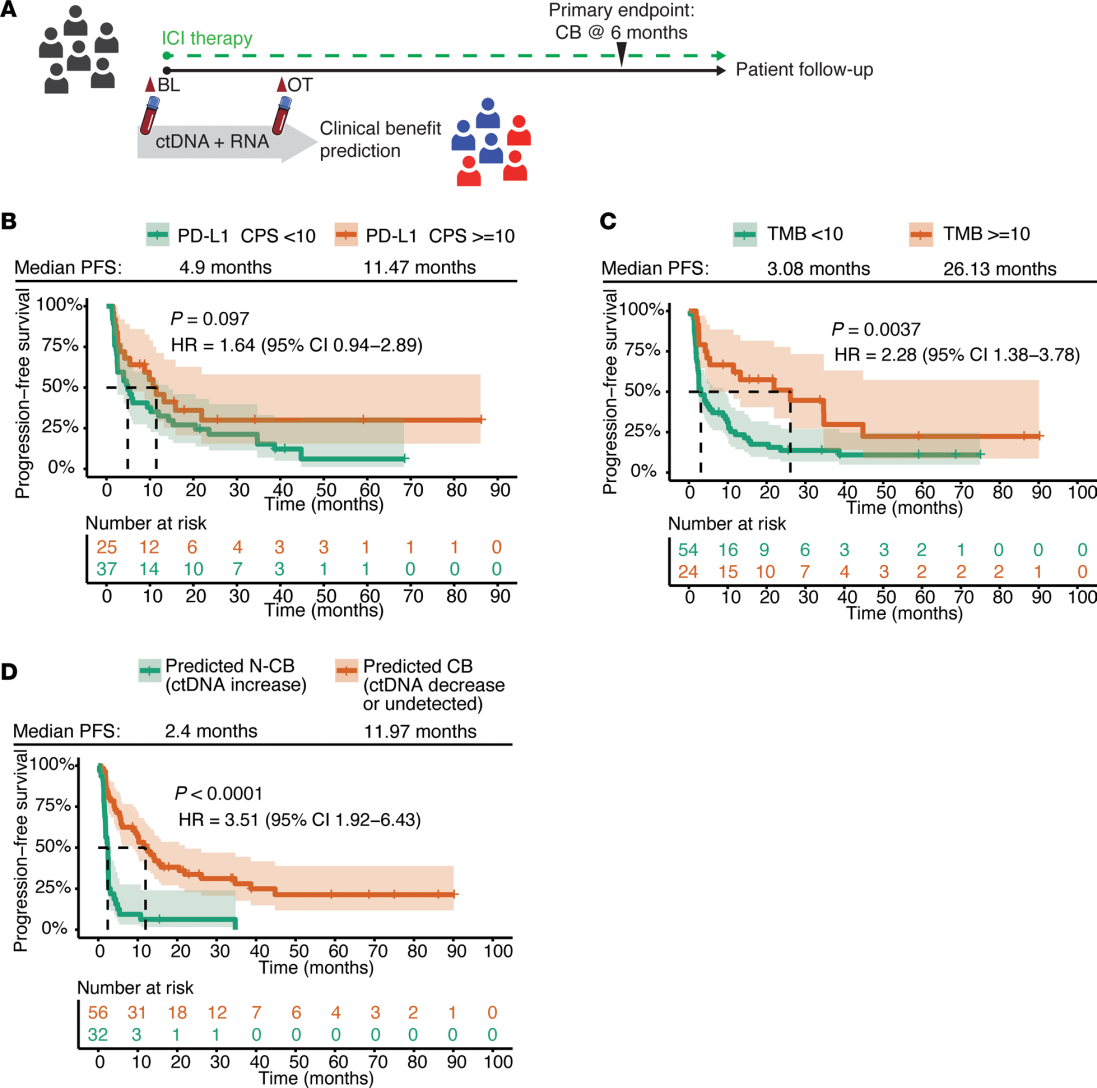
Circulating tumor DNA (ctDNA) dynamics predicts clinical benefit (CB) to immune checkpoint inhibitor (ICI) therapy in metastatic urothelial cancer (mUC) patients. (**A**) Sample collection and analysis schematic: mUC patients were treated with an ICI (pembrolizumab, nivolumab, or avelumab) until disease progression. Blood was collected at baseline (BL, before cycle 1) and on-treatment (OT, after 2–6 weeks) for both ctDNA and RNA analysis. The primary endpoint was CB. This was defined as progression-free survival (PFS) for at least 6 months. (**B**) Kaplan-Meier (KM) curve comparing the PFS of patients with PD-L1–positive tumor (orange curve, PD-L1 combined positive score < 10) and patients with PD-L1–negative tumor (green curve, PD-L1 combined positive score < 10). (**C**) KM curve comparing the PFS of patients with a high tumor mutational burden (TMB) (orange curve, TMB ≥ 10 mutations/Mb) and TMB low patients (green curve, TMB < 10 mutations/Mb). (**D**) KM curve comparing the PFS of ctDNA-based patient stratification. The predicted CB population (orange curve) contains patients who had a decrease in ctDNA fraction from BL to OT or undetected at both time points. The predicted non-clinical benefit (N-CB, green curve) population contains patients where the ctDNA fraction increased from BL to OT or was stable. *P* values were determined by a Mantel-Haenszel test. HR, hazard ratio; CI, confidence interval.

To longitudinally and noninvasively monitor response to ICIs and to discover biomarkers predictive of CB, we collected blood liquid biopsies (LBx) from a total of 93 mUC patients treated with pembrolizumab (*n* = 72), nivolumab (*n* = 7), or avelumab (*n* = 14) (Figure 1A, Supplemental Figure 1A, and Table 1; supplemental material available online with this article; https://doi.org/10.1172/jci.insight.186062DS1). Specifically, BL blood LBx were collected before ICI therapy initiation, while OT LBx were collected after cycle 1 or 2 (2–6 weeks). LBx samples were used for ctDNA and bulk whole-blood RNA-seq analysis (see Methods for details). We collected paired BL and OT ctDNA data for 88 patients and RNA-seq data for 79 patients, 74 of whom had both ctDNA and RNA data. Moreover, archival tumor tissue (formalin-fixed, paraffin embedded [FFPE]) was used to determine PD-L1 combined positive score (CPS) (*n* = 62) and TMB (*n* = 78) (Table 1). Of note, for RNA-seq analysis and modeling, patients were distributed in separate discovery, testing, and validation cohorts for optimal data analysis (Supplemental Figure 1A). Clinical endpoint was clinical benefit at 6 months, defined as radiological and clinical progression-free survival (PFS) at or beyond 6 months from treatment initiation (Supplemental Figure 1B). Out of the 93 patients included, 42 patients experienced CB and 51 did not (N-CB). Clinical characteristics are described in Table 1.

### ctDNA profiling outperforms conventional tumor biomarkers for prediction of N-CB in ICI-treated mUC patients.

High TMB and PD-L1 expression in the tumor have previously been associated with CB to ICIs (16, 17). We, therefore, assessed whether these 2 tumor biomarkers could help stratify the cohort into patients with or without CB (Figure 1, B and C). High PD-L1 expression was defined as a CPS of 10 or higher, in line with what is used in the clinic to select cisplatin-ineligible mUC patients for first-line ICIs. There was a weak trend, but no significant association, between a CPS of 10 or higher and longer PFS (Figure 1B). High TMB, defined as 10 mutations or more per megabase, was significantly associated with improved PFS (Figure 1C), but low TMB had only 80% sensitivity and 43% specificity to predict N-CB. These results indicate that conventional tumor biomarkers only have partial ability to predict CB to ICIs in mUC and that more accurate biomarkers are needed.

We previously demonstrated that decreases in ctDNA after 3–6 weeks show high specificity and moderate sensitivity for predicting CB to ICIs in a subset of patients of the presented mUC cohort (15). We expanded our previous cohort for the current analyses to a total of 88 patients. Patients were categorized into 2 groups based on their ctDNA dynamics. Patients with a ctDNA increase or stable from BL to OT were predicted to have N-CB (*n* = 32), while patients with a ctDNA decrease from BL to OT (*n* = 45) or undetectable ctDNA at both time points (*n* = 11) were predicted to have CB. Patients with predicted CB had significantly longer PFS compared with those with predicted N-CB (Figure 1D). The ctDNA-based model showed 59% sensitivity and 92% specificity to detect N-CB patients (true positive cases) (Supplemental Figure 1C). Additionally, a larger decrease in ctDNA level correlated with an extended time to progression (Supplemental Figure 1D). Interestingly, ctDNA-based predictions partially correlated with PD-L1 CPS- and TMB-based stratification (Supplemental Figure 1E). Altogether, these data support the potential of ctDNA to predict N-CB, which outperforms the conventional tumor biomarkers PD-L1 and TMB.

### Longitudinal immunotranscriptome analyses reveal biologically relevant changes in patients with CB to ICIs.

While early increases in ctDNA were highly specific for N-CB, only 59% of the patients with N-CB were identified with this approach, possibly related to the fact that ctDNA levels do not capture immune activity. We reasoned that the addition of a second approach capturing host immune response using LBx might improve biomarker accuracy compared with the ctDNA-only approach. We, therefore, decided to investigate whether we could use the peripheral blood immunotranscriptome for early response prediction to ICIs. To investigate this, patients were distributed into independent discovery, testing, and validation cohorts (Supplemental Figure 1A). Specifically, the first cohort was used for biomarker discovery and model training, the second for independent model testing and optimization, and the third for final, blinded, model validation.

**Figure 2:**
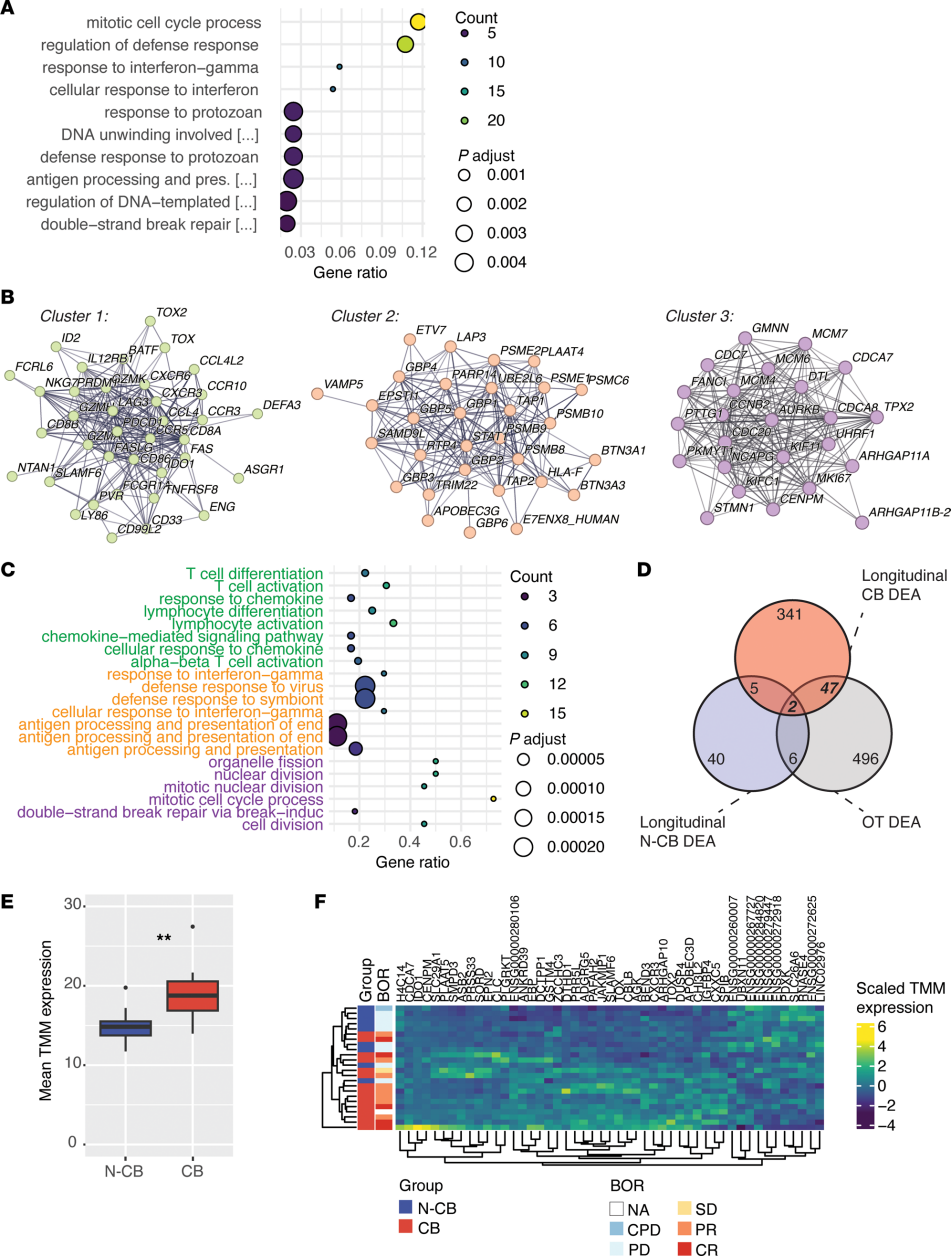
Blood immunotranscriptome dynamics in CB patients reveal the biological mode of action of early response to ICIs. (**A**) Over-representation analysis (ORA) performed on the upregulated differentially expressed genes at OT (edgeR fold-change > 0) found by differential expression analysis (DEA) comparing paired BL to OT samples of CB patients (longitudinal CB DEA). The top enriched gene ontology biological processes (GO BPs) are shown (based on an enrichment-adjusted *P* value *≤* 0.05), highlighting pathways upregulated at OT. (**B**) Largest gene clusters identified by STRING analysis of all DEGs in the longitudinal CB DEA. Each node represents 1 gene and each segment an interaction defined by STRING analysis. (**C**) ORA performed on the genes included in the clusters showed in **B**. The top GO BPs are shown (based on an enrichment-adjusted *P* value *≤* 0.05, green terms are associated to cluster 1, orange terms with cluster 2, and violet terms with cluster 3). (**D**) Venn diagram showing the DEGs intersect between the longitudinal CB DEA (395 DEGs), the DEA comparing paired BL to OT samples of N-CB patients (longitudinal N-CB DEA, 53 DEGs), and the DEA comparing CB to N-CB patients at the OT time point (OT DEA, 551 DEGs). The 49-gene intersect between the longitudinal CB DEA to the OT DEA is highlighted. (**E**) Box-and-whisker plot comparing the mean expression of the 49-gene set highlighted in **D** in the N-CB and CB patient group at the OT time point. Gene expression is defined for each patient by the mean of the trimmed mean of M values (TMM) for each gene in the 49-gene set. (**F**) Expression heatmap and hierarchical clustering of the 49-gene set in N-CB and CB patients at the OT time point. Columns and rows are hierarchically clustered. Patient group and best overall response (BOR) are annotated per row. NA, not annotated; CPD, clinical progressive disease; PD, progressive disease; SD, stable disease; PR, partial response; CR, complete response. **P < 0.01 by Wilcoxon’s test.

For biomarker discovery, we first explored the longitudinal changes in gene expression in the CB patients in the discovery cohort (*n* = 29). We performed differential gene expression analysis (DEA) comparing paired BL and OT samples of CB patients (longitudinal CB DEA) followed by pathway analysis of the differentially expressed genes (DEGs) (Supplemental Figure 2A). Interestingly, among the over-representation analysis (ORA) of upregulated processes at OT, we found several pathways related to cell cycle regulation and adaptive immune system signaling (including antigen presentation and interferon-γ signaling) (Figure 2A and Supplemental Figure 2B), while no significantly enriched pathways could be identified by analyzing the downregulated DEGs. These results were confirmed by STRING network analysis of all DEGs, which identified 3 highly interacting gene clusters related to T cell activation, interferon-γ signaling, and cell cycle regulation (Figure 2, B and C). The 3 gene clusters consisted mainly of genes that were upregulated during treatment (Supplemental Figure 2C) and included genes that have previously been associated with response to ICIs, such as *PDCD1* (PD-1), granzymes, and *MKI67* (18–20). Moreover, STRING network analysis enabled the identification of biologically relevant genes downregulated at OT, which included several myeloid cell–specific genes (*PVR*, *CD33*, *ENG*, *LY86*, *CD86*, *TNFRSF8*, and *ASGR1*) belonging to STRING network cluster 1.

To identify the genes that discriminate CB and N-CB patients, we compared the longitudinal CB DEA DEGs to DEGs that are differentially expressed between BL and OT time points in N-CB patients (longitudinal N-CB DEA) and DEGs that were differentially expressed between OT samples of CB and N-CB patients (OT DEA) (Figure 2D and Supplemental Figure 2D). Interestingly, the longitudinal N-CB DEA (BL vs. OT time points) only revealed few DEGs (53 genes), of which only 7 genes were shared with the longitudinal CB DEA DEGs. By contrast, the OT DEA showed a larger overlap with the longitudinal CB DEA (49 genes), showing that these genes not only are differentially regulated after treatment in the CB population, but also discriminate the CB and N-CB groups at the OT time point. Functional analysis of the 49-gene intersect revealed pathways related to T cell tolerance (Supplemental Figure 2E). Altogether, the functional analysis of the longitudinal CB DEA highlighted multiple gene sets with the potential of discriminating the CB from the N-CB group.

We subsequently investigated whether the identified 49-gene set (Figure 2D) and the 3 STRING network clusters (Figure 2B) were able to separate the CB and N-CB patients. Specifically, we assessed the mean gene expression at OT in the CB and N-CB groups (Figure 2E and Supplemental Figure F) and patient clustering based on the gene expression at OT (Figure 2F and Supplemental Figure 2G). Interestingly, we found that the 49-gene set had significantly higher expression in the CB group and could separate the 2 populations by heatmap analysis (Figure 2, E and F), while STRING cluster 1 to 3 gene sets could not achieve such separation (Supplemental Figure 2, F and G). These results indicated that immunotranscriptomic data from whole blood can detect biologically relevant signals of an early peripheral response to ICIs. Moreover, the identified genes have potential to stratify the CB and N-CB populations.

### Immunotranscriptome data and machine-learning approaches allow developing robust models for early response prediction to ICIs.

**Figure 3:**
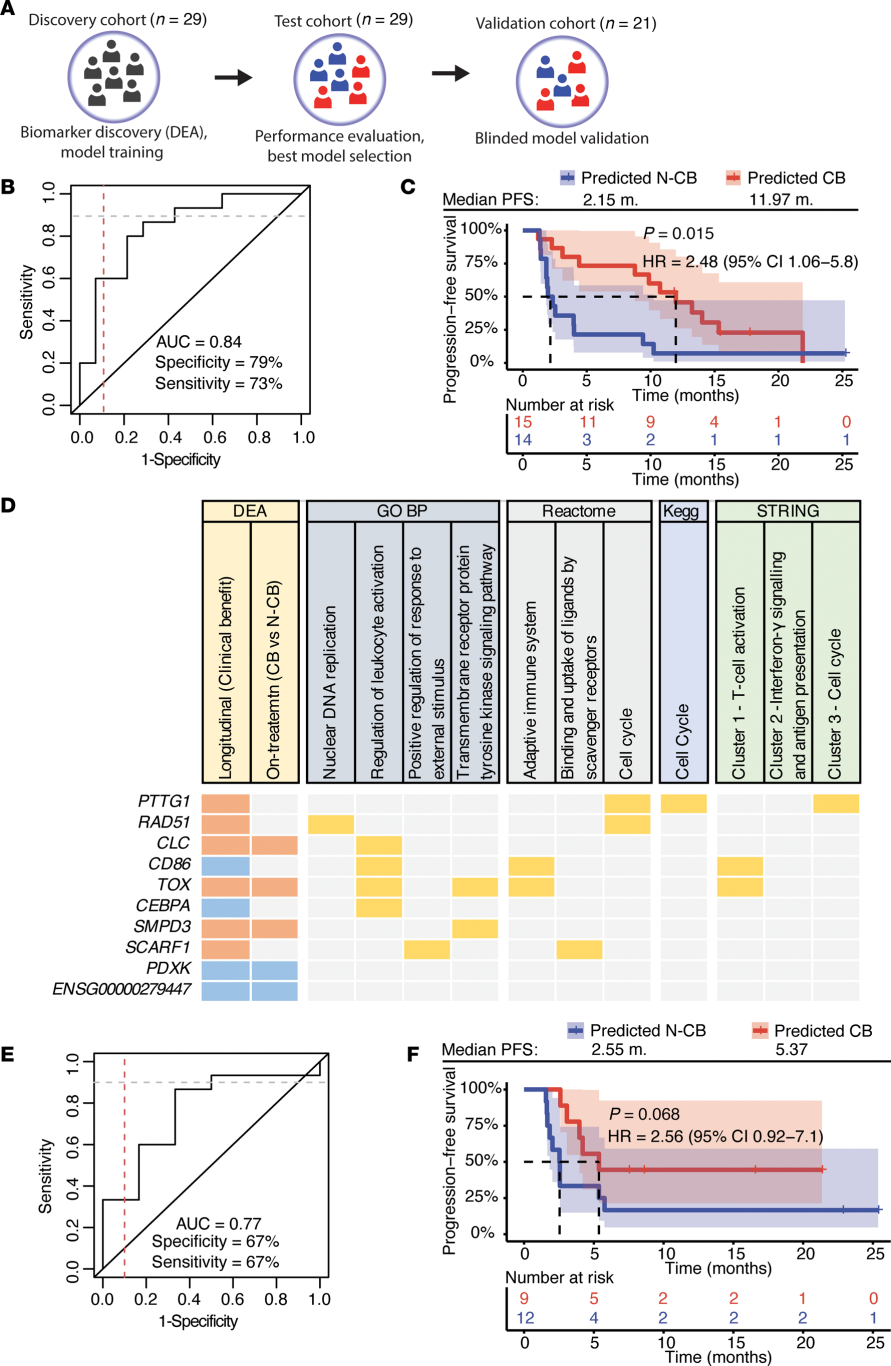
Blood-based immunotranscriptome predictive model forecasts CB in an independent cohort. (**A**) Modeling approach schematic: Biomarker discovery was performed in the discovery cohort (patients with paired BL and OT RNA-seq data, *n* = 29) by DEA. Model training was performed in the same cohort by multiple iterations of random features reduction of the biomarker/gene list, followed by model testing in the independent test cohort (patients with paired BL and OT RNA-seq data, *n* = 29). The best CB predictive model was selected by area under the curve (AUC) ranking of each model receiver operating characteristics (ROC) curve and by ranking the difference in median PFS between the predicted CB and N-CB groups in the test cohort (*n* = 29). Last, the best performing model was validated in the validation cohort (patients with paired BL and OT RNA-seq data, *n* = 21). (**B**) ROC curve showing model performance of the best performing model in the independent test cohort (*n* = 29). Specificity is calculated with respect to CB patients (true negative cases), while sensitivity to N-CB (true positive cases). (**C**) Kaplan-Meier (KM) curve comparing the PFS of model-based predicted CB population (red) and predicted N-CB population (blue) in the independent test cohort (*n* = 29). (**D**) Attention map contextualizing the biology of the 10 genes used to craft the model shown in **B** and **C** showing in which DEA the genes were identified. The genes have also been mapped to a selection of significantly enriched pathways of different ontologies in the longitudinal CB DEA (enrichment-adjusted *P* ≤ 0.05) and to the STRING network clusters shown in Figure 2B. Genes included in the DEGs of the longitudinal CB DEA or the OT DEA are highlighted in orange (upregulated, based on edgeR FC ≥ 0) or in blue (downregulated, based on edgeR FC < 0). Genes associated with enriched pathways or STRING clusters are highlighted in yellow. (**E**) ROC curve showing model performance assessment in the independent blinded validation cohort (*n* = 21). Specificity is calculated with respect to CB patients (true negative cases) and sensitivity to N-CB (true positive cases). (**F**) KM curve comparing the PFS of RNA model–based predicted CB population (red) and N-CB population (blue) in the independent blinded validation cohort (*n* = 21). *P* values were determined by a Mantel-Haenszel test. HR, hazard ratio (predicted CB population as reference); CI, confidence interval.

To develop predictive models of CB to ICIs, the DEGs identified in the longitudinal CB DEA of the discovery cohort were used as input to generate multiple models, using several iterations of biomarker subset selection (Figure 3A and Supplemental Figure 1A) (see Methods for details). Subsequently, the best performing immunotranscriptome model (RNA model) was selected based on predictions in the independent test cohort. This final RNA model, comprising 10 genes, was selected based on area under the curve (AUC) ranking of the receiver operating characteristics (ROC) curve for predicting CB to ICIs and by ranking the difference in median PFS between the predicted CB and N-CB groups (Figure 3, B and C, and Supplemental Figure 3, A and B). The RNA model showed high performance in both the independent test cohort (73% sensitivity at 79% specificity, AUC = 0.84, *n* = 29) and discovery cohort (92% sensitivity at 71% specificity, AUC = 0.86, *n* = 29). We then investigated the biological role of the 10 genes selected by machine learning–based feature reduction and used in the RNA model by mapping them to an attention map contextualizing the underlying biology (Figure 3D). We found that 8 out of 10 genes (*PTTG1*, *RAD51*, *CLC*, *CD86*, *TOX*, *CEBPA*, *SMPD3*, and *SCARF1*) could be contextualized to relevant biological functions of early response to ICIs previously identified by ORA, such as T cell activation and proliferation, while 2 out of 10 (*PDXK* and a noncoding RNA) genes were not associated with previously identified pathways, but both belonged to the 49-gene set (Figure 2D). Interestingly, when using either the STRING gene clusters or the 49-gene set to develop predictive models, the performance in the test cohort was inferior to the 10-gene RNA model (Supplemental Figure 3C). Also, the 10-gene panel did not show any overlap with a selection of literature-based tumor-derived signatures that have been shown to correlate with CB to ICIs in other studies (Supplemental Figure 3D) (21–23), although some of these genes were among the longitudinal DEA DEGs. Of note, models based on these literature-based signatures showed low performance in our cohorts (Supplemental Figure 3E). Lastly, we validated the model performance in a small blinded validation cohort (*n* = 21). The RNA model achieved an AUC of 0.77 (67% sensitivity at 67% specificity, *n* = 21) (Figure 3E) and there was a nonsignificant trend toward longer PFS in the predicted CB group (Figure 3F). To summarize, machine learning–based approaches for biomarker selection and modeling identified the most central RNA biomarkers in whole blood and showed high accuracy in the discovery and testing cohort for predicting CB.

### Multimodal modeling with ctDNA- and RNA-based biomarkers boosts model performance and leads to accurate prediction of N-CB in the independent validation cohort.

**Figure 4:**
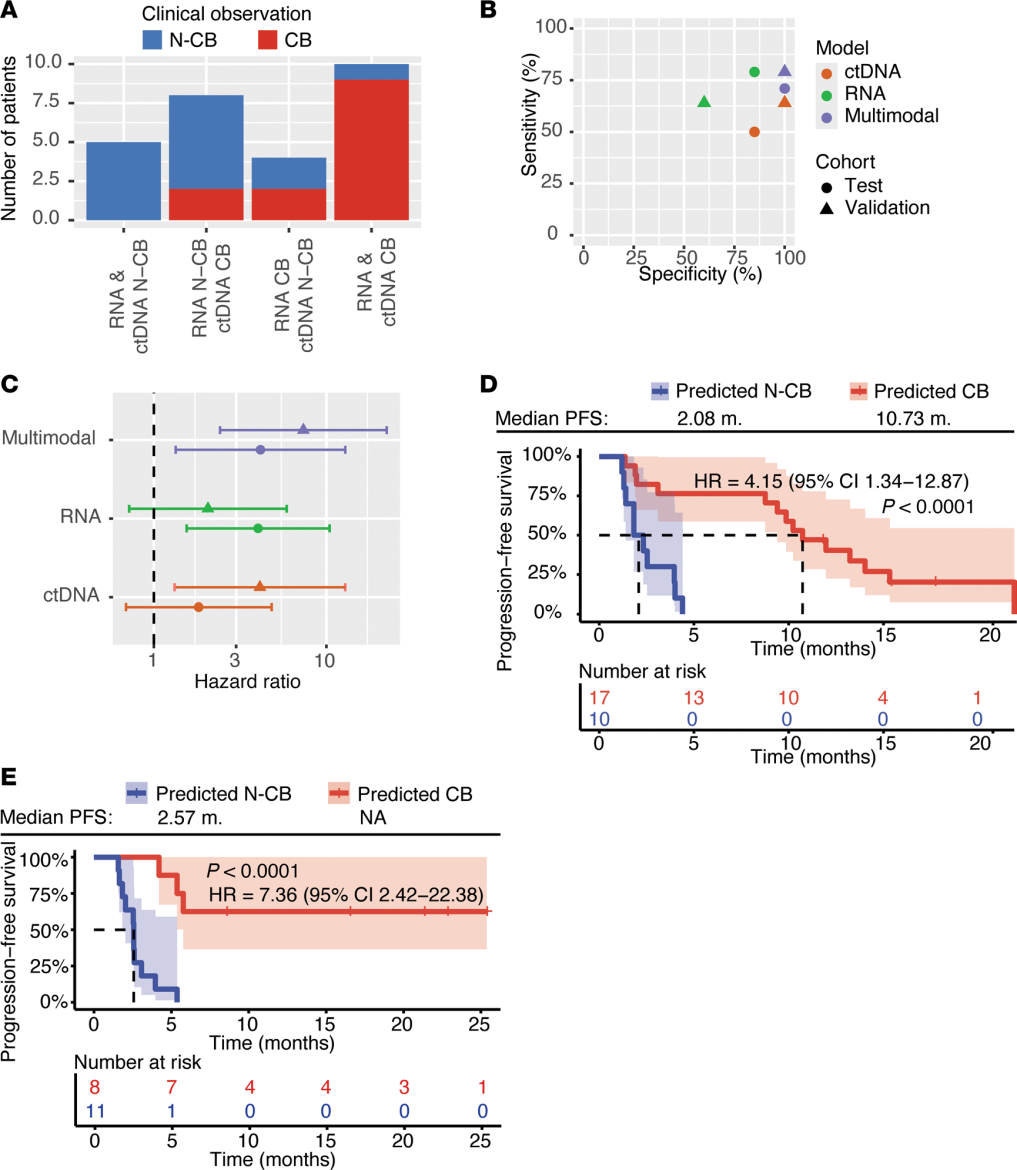
Integration of ctDNA- and RNA-based biomarkers boosts the performance of a multimodal model in an independent blinded validation cohort. (**A**) Prediction comparison: Patients of the independent test cohort (*n* = 27, where both RNA-seq and ctDNA data were available) were categorized based on the RNA and ctDNA model predictions, highlighting convergent or divergent readouts by the 2 approaches. Column color coding reflects the actual CB group defined by clinical assessment (red = CB, blue = N-CB). (**B**) Model performance comparison of the different model approaches (ctDNA model in orange, RNA model in green, multimodal model in violet) in the independent test cohort (circles, *n* = 27, where both RNA-seq and ctDNA data were available) and blinded validation cohort (triangles, *n* = 19, where both RNA-seq and ctDNA data were available). (**C**) Hazard ratio (HR) for PFS of the 3 modeling approaches used for patient stratification (ctDNA model in orange, RNA model in green, multimodal model in violet) in the independent test (circles, *n* = 27, where both RNA-seq and ctDNA data were available) and blinded validation cohorts (triangles, *n* = 19, where both RNA-seq and ctDNA data were available). The bars represent the confidence of interval for each HR. The dashed line represents HR = 1. (**D** and **E**) Kaplan-Meier curves comparing the PFS of the multimodal model–based predicted CB population (red) and N-CB population (blue) in (**D**) the independent test cohort (*n* = 27, where both RNA-seq and ctDNA data were available) and (**E**) in an additional blinded and independent validation cohort (*n* = 19, where both RNA-seq and ctDNA data were available). *P* values were determined by a Mantel-Haenszel test. HR, hazard ratio (predicted CB population as reference); CI, confidence interval.

Emerging evidence suggests that combining biomarkers from dissimilar sources can exponentially improve model performance (24, 25). We, therefore, hypothesized that we could improve predictions by integrating RNA- and ctDNA-based readouts in one model. When comparing the predictions of the standalone ctDNA- and RNA-based models in the test cohort (Figure 4A and Supplemental Figure 4A), we found that models showed discordant predictions for a significant proportion of patients (12 out of 27). We then reasoned that the fixed cutoffs used in the standalone models might influence wrong readout occurrence. Indeed, false-positive and false-negative cases of both the RNA and the ctDNA model had a prediction probability and, respectively, a ctDNA ratio close to the cutoff values (Supplemental Figure 4, B and C). Therefore, we developed a multimodal model in the test cohort where the final prediction was based on a cutoff range of combined ctDNA ratio and RNA-based prediction probability (see Methods for details). The multimodal model was then validated in the independent validation cohort. The multimodal model showed superior performance compared with the standalone approaches (Figure 4B), reaching 71% sensitivity at 100% specificity in the test cohort, and 79% sensitivity at 100% specificity in the independent blinded validation cohort. Consequently, the multimodal approach allowed for a superior stratification of the predicted CB and N-CB groups (Figure 4, C–E). Thus, this multimodal analysis appears a promising tool for early response prediction to ICIs.

## Discussion

In this study, we performed ctDNA and whole-blood immunotranscriptome analyses to identify biomarkers for early response prediction to first- and second-line and maintenance ICIs in patients with mUC. We confirmed that early changes in ctDNA levels are associated with CB to ICIs, with early increases in ctDNA levels being highly specific for N-CB. We hypothesized that peripheral blood immunotranscriptome analyses might be a valuable complementary approach to ctDNA and indeed found it to be a promising tool for early response monitoring in mUC. In patients with CB to ICIs, pathways related to cell cycle regulation, T cell activation, antigen presentation, and interferon-γ signaling were upregulated already 3 weeks after the first anti–PD-(L)1 infusion. These changes were specific for patients with CB, which enabled the generation of a 10-gene model to predict CB based on whole-blood immunotranscriptome data with high accuracy. A multimodal model incorporating both ctDNA and immunotranscriptome predictions showed superior performance compared with both standalone predictions and conventional biomarkers (PD-L1 and TMB) and demonstrated a sensitivity of 79% and specificity of 100% for prediction of N-CB in an independent blinded validation cohort.

There are currently no clinically applicable biomarkers to identify patients that derive benefit from ICIs. In current practice, ICIs are usually continued for at least 12 weeks, at which point the first radiological response evaluation is performed. Clinically stable patients with suspected progression after the first scan according to iRECIST may continue treatment to avert treatment discontinuation in patients with pseudo-progression or a delayed response (26). Early response biomarkers would facilitate the early identification of patients without CB, thereby limiting unnecessary costs and toxicities. Additionally, the use of early response biomarkers may improve clinical outcomes by facilitating an early treatment switch or treatment intensification in patients that do not benefit from ICI monotherapy. Early OT biomarkers would, therefore, be of great value in the clinic.

In this study, we observed that changes in ctDNA levels during ICI treatment are associated with clinical outcome. Others have described comparable associations between ctDNA kinetics and clinical outcome following ICIs in multiple cancer types, but the optimal cutoff remains to be elucidated (14, 27, 29–32). Raja et al. showed a relationship between increases in ctDNA fraction and disease progression in 28 mUC patients treated with ICIs, similar to our data (27). Powles et al. recently presented ctDNA data of the phase III KEYNOTE-361 trial. Changes in ctDNA fraction during the first 3 weeks of pembrolizumab were smaller than during chemotherapy, but showed a stronger association with clinical outcomes. Additionally, patients with a large reduction in ctDNA after 3 weeks, defined as a reduction above the median across both treatment groups, demonstrated higher objective response rates and better OS (14). In our study, we chose to split the cohort into patients with or without a ctDNA increase in order to detect patients with N-CB with high certainty. This way, clinicians and patients could confidently decide on an early treatment switch or intensification without risking halting an effective treatment. Nevertheless, when using another cutoff it might also be possible to use ctDNA to detect long-term responders with high specificity, as observed in our dataset (Supplemental Figure 1D).

To improve early response prediction based on ctDNA data alone, we reasoned that the addition of a second approach that reflects immunological activity might improve our predictions. To enable noninvasive monitoring of peripheral immune activity, we investigated the potential of whole-blood immunotranscriptome analyses for the early identification of response to ICIs in mUC and were able to generate a 10-gene model to predict CB based on peripheral blood immunotranscriptome data with high accuracy.

Although our immunotranscriptome analyses were performed on bulk RNA-seq data, we found several biologically relevant pathways to be upregulated during the first weeks of therapy in patients with CB to ICIs, including pathways involved in cell cycle regulation, T cell activation, and antigen presentation and interferon-γ signaling, confirming that whole-blood immunotranscriptome data are a reliable source to detect ICI-related changes in peripheral blood. Interestingly, we found some immune-related genes that have previously been associated with response to ICIs in other studies using tumor biopsies (21–23), highlighting the capability of our LBx approach to detect parallel gene dysregulation in the blood. Of note, while several studies have investigated the relationship between baseline tissue transcriptome and response to ICIs, studies using peripheral blood transcriptome data are scarce. Interestingly, a recent study by Richard et al. showed that genes associated with immune cell activation are overexpressed in baseline samples of mUC patients responding to durvalumab, whereas patients with progressive disease overexpressed genes of immune cell inhibition (33). These authors, however, did not study OT changes in whole blood RNA. While data on early changes in peripheral blood transcriptome in patients treated with ICIs are lacking, our findings are in line with previous flow cytometry and single-cell sequencing studies, demonstrating proliferation of (activated) T cells during the early phase of ICI therapy in patients that benefit from ICIs (18, 19, 34).

Whereas longitudinal ctDNA dynamics reflect changes in tumor burden and biological activity, whole-blood immunotranscriptome dynamics reflect early, systemic adaptations in immune cell activity and proliferation. We, therefore, hypothesized that a multimodal model capturing both ctDNA and whole-blood immunotranscriptome predictions might outperform the standalone approaches. Our multimodal model indeed showed superior performance compared with the standalone approaches. While the ctDNA standalone approach had a sensitivity and specificity of 64% and 100% and the RNA standalone approach 67% and 67% in the independent validation cohort, the multimodal model reached 79% sensitivity and 100% specificity.

While our multimodal model will need further validation before it can be implemented in the clinic, our model shows promise as a noninvasive biomarker test for the early detection of N-CB to ICIs. The high specificity will allow clinicians and patients to confidently decide on an early treatment switch without risking halting an effective treatment. While it would be acceptable to miss some patients with N-CB, we should aim to further optimize the sensitivity of our test in subsequent studies. One possibility to optimize test performance is the incorporation of a second, early, OT sample. Our previous ctDNA study (15) analyzed 20 patients with both 3-week and 6-week samples; 2 patients with N-CB showed a ctDNA decrease at 3 weeks, but an increase at 6 weeks. On the other hand, 1 patient with CB showed a rise in ctDNA at 3 weeks and then a decrease compared with the 3-week time point at 6 weeks. These data suggest that more insight into early dynamics of ctDNA might further improve the performance of our test. Another way to improve our test is by incorporating additional mUC-associated genes in the ctDNA panel (e.g., *KMT2D* and *KDM6A*) (15). It is possible that a few patients with undetectable ctDNA in our study had false-negative results and were incorrectly categorized, negatively influencing the accuracy of our ctDNA prediction. Further optimization of our ctDNA panel could limit the number of patients with false-negative ctDNA testing.

Our multimodal model based on whole-blood immunotranscriptome and ctDNA data shows promise as a noninvasive blood-based biomarker test for early identification of N-CB to ICIs in mUC. Interestingly, the model obtained accurate predictions in both patients treated with first- or second line ICIs as well as in patients treated with avelumab maintenance, emphasizing the robustness of the test. Yet, this study also has some limitations. First, the study cohorts were small, particularly the number of patients with paired ctDNA and RNA data in the independent validation cohort (*n* = 19). Validation of our multimodal model in larger cohorts is needed before it can be implemented in the clinic. Another limitation is that the use of ICI monotherapy may decline in the near future due to changes in the treatment landscape of mUC. Nevertheless, we anticipate that ICI monotherapy will continue to be an important treatment modality for frail or elderly patients with mUC because of the high toxicity associated with combination therapies. Additionally, it would be very interesting to test whether our multimodal biomarker approach can be used in the first-line setting to limit the use of intensive combination therapies to patients that do not durably benefit from ICI monotherapy. For instance, patients could receive pembrolizumab monotherapy in the first-line mUC setting and could then escalate to pembrolizumab-EV if the multimodal test predicts that the patient is not responding. This strategy may particularly be of interest in patient subgroups that derive increased benefit of ICI monotherapy, such as those with lower ctDNA fractions or those with lymph node–only disease (14, 35).

While the current study tested the predictive value of early ctDNA and whole-blood RNA kinetics in patients receiving ICI monotherapy, it would also be of interest to test these biomarkers in patients receiving ICI-containing combination strategies, such as EV or cisplatin-gemcitabine. Not all patients derive benefit from addition of an ICI, and response patterns from longitudinal assessment might distinguish those that derive benefit from combination therapy, or EV or ICI alone.

In conclusion, whole-blood immunotranscriptomics provides a promising tool for early response prediction to ICIs in mUC, particularly when used in a multimodal model together with changes in ctDNA levels. Results of our multimodal analyses should be validated in clinical trials to confirm that the test can be used to improve clinical outcomes of mUC patients.

## Methods

### Sex as a biological variable.

Both female and male patients were included. In this study, sex was not considered as a biological variable.

### Patients.

This Dutch, multicenter study included 93 patients with mUC who were treated with anti–PD-(L)1 between 2017 and 2023. Patients were treated with nivolumab or pembrolizumab in the first- or second-line mUC setting or with maintenance avelumab following response or stable disease to platinum-based chemotherapy. Patients with measurable disease were evaluated according to RECIST1.1 (36). Clinical endpoint was CB at 6 months, defined as radiological and clinical PFS at 6 months, which was previously demonstrated to show a better correlation with OS in mUC than objective response (37). Patient demographics are reported in Table 1.

### Blood collection and processing.

Blood was drawn prior to the first 3 cycles of anti–PD-(L)1 therapy (i.e., at 0, 2, and 4 weeks for nivolumab and avelumab and at 0, 3, and 6 weeks for pembrolizumab). At these time points, a complete blood cell count was performed as part of routine clinical care. In addition, blood was collected in a PAXgene Blood RNA tube for whole-blood RNA analyses (BD Biosciences) and in 3, 10 mL EDTA or cell-free DNA (cfDNA) collection tubes (Roche) for ctDNA analyses. PAXgene tubes were stored at –80°C until RNA purification. EDTA and cfDNA tubes were processed as previously described (15). The BL sample and the earliest OT sample available were used for analyses.

### TMB and PD-L1.

Tumor tissue for molecular analysis and PD-L1 staining was obtained from diagnostic biopsies obtained in routine clinical practice. The PD-L1 staining was performed on FFPE tissue sections using antibodies against 22C3 (PharmaDx kit, DAKO Agilent, GE006) or E1L3N (Cell Signaling Technology, 13684S). CPS was calculated by dividing the number of stained cells expressing PD-L1 (tumor cells, tumor-associated lymphocytes, and macrophages) by the total number of viable tumor cells, multiplied by 100, taking into account at least 200 viable tumor cells, not adjacent to necrotic areas. A CPS of 10 or higher was considered positive.

Tumor sequencing data were generated utilizing different sequencing platforms: whole-genome sequencing (WGS), whole-exome sequencing (WES), TruSight Oncology 500 (TSO500), Foundation Medicine T7 assay (CLIA: 22D2027531), single molecule Molecular Inversion Probe panel (PATHv3D), and/or the ctDNA_NGSv1 targeted sequencing panel (15). WGS, WES, and TSO500 data were used to determine nonsynonymous (nsTMB). An nsTMB of 10 or more mutations per megabase was considered high.

### ctDNA.

ctDNA analyses were performed in 88 patients, 53 of whom were included in a prior publication (15). Only patients with paired BL and OT samples who were evaluable for the clinical endpoint were included in the current analyses.

ctDNA workup and downstream analysis were performed as previously described (15). In short, cfDNA was isolated from blood plasma (median 5.6 mL, IQR 5–8 mL) using the QIAamp Circulating Nucleic Acid kit (Qiagen). White blood cell (WBC) DNA was isolated using a QIAamp DNA Mini Kit (Qiagen). A maximum of 50 ng cfDNA (median 50 ng, IQR 37–50 ng) and 50–80 ng of mechanically sheared WBC DNA were used for targeted sequencing using an in-house developed and validated 117 kb targeted sequencing panel (NEN-EN-ISO 15189+C11:2015) (15). Libraries were generated using the TWIST Library Preparation Kit (TWIST Biosciences) in combination with xGen dual index unique molecular identifier (UMI) adapters (Integrated DNA Technologies) or TWIST UMI adapters (TWIST Biosciences). Libraries were paired-end sequenced on a NovaSeq 6000 platform (Illumina). Reads were mapped to hg19 and deduplicated using the read-specific UMI information (fgbio). Unique reads that not met fgbio quality parameters and/or based on less than 2 UMI reads (singletons) were only kept for variant detection in the *TERT* protomer region and copy number variant (CNV) detection.

Somatic variants were called using Genomic Analysis Toolkit (GATK) Mutect2 (version 4.1.5.0) based on previously described filter criteria (15). Variants with at least 5 supporting variant reads and greater than 0.1% variant allele fraction (VAF) were selected for downstream analysis. Additionally, patient-specific cfDNA variants and, if available, tumor variants (evaluation of nonsynonymous tumor variants with a minimal read depth of 10, *n* = 59 patients) were evaluated in the patient-matched BL and OT cfDNA sequencing data. For this dependent calling, the variant in the matched BL or OT sample had to be supported by at least 3 variant reads, the VAF signal had to be at least 20 times higher than the average VAF of 22 control cfDNA samples, and at least 3 times higher than the patient-matched WBC sample for that specific nucleotide change (if available).

CNV detection was performed as previously described using both the relative coverage and the median allele fraction (MAF) divergence from heterozygosity. Copy number loss was defined as relative coverage of −0.3 or less, or relative coverage of −0.1 or less and a MAF of 0.6 or greater. Copy number gain was defined as a relative coverage of 0.3 or greater, or 0.1 or greater and MAF of 0.6 or greater.

ctDNA fraction was determined by using the somatic mutation with highest VAF in a non-amplified region corrected for loss of heterozygosity or using the MAF deviation from heterozygosity of germline single nucleotide polymorphisms in genes with a single-copy loss (15, 38). ctDNA fractions were converted to ctDNA copies per mL plasma (total cfDNA concentration multiplied by 303). To incorporate technical uncertainty and biological variability of ctDNA levels, lower and higher limits were estimated as previously described (15).

OT changes were dichotomized into increase versus no increase, based on changes in ctDNA copies/mL. Patients with an increase were predicted to not have CB (N-CB), whereas patients without an increase during treatment were predicted to have CB. Patients with undetectable ctDNA in both the BL and OT sample were categorized as no increase/predicted-CB since low BL ctDNA levels are considered a prognostically favorable sign (14). ctDNA-based specificity and sensitivity was calculated for the full ctDNA cohort.

### Whole-blood RNA-seq.

Whole-blood RNA-seq was performed in 79 patients with paired samples. Additionally, 2 patients with OT samples only were used for DEA. Total RNA was extracted from whole blood using the PAXgene blood miRNA kit (Qiagen). RNA quantity was determined using a Qubit (Thermo Fisher Scientific). RNA quality was assessed on a TapeStation 4200 (Agilent Technologies). Per sample, at least 200 ng of total RNA was used for library preparation. RNA samples were treated for globin RNA depletion with the QIAseq FastSelect RNA Removal kit (Qiagen). Library preparation was performed after isolation of poly-A RNA by means of NEBNext poly(A) mRNA magnetic isolation module and then, setup of directional RNA libraries by means of NEBNext Ultra II directional RNA library prep kit in combination with NEBNext multiplex oligos for Illumina Set 1, Set 2, and Set 3 was performed (NEB). Library quality control was done by using Dual AmpureXP cleanup for complete adapter dimer removal, and a verification of adapter dimer removal with a TapeStation 4200.

All libraries were pooled by equal volume and a test sequencing run was done on an iSeq 100 (Illumina) to determine the content of each library and adjust the final pool. Sequencing was performed on an Illumina NovaSeq 6000, 3 lanes of S4 flow cell, paired-end 150-bp configuration with an expected output of 800 Gb per lane or ca. 2,600M paired-end (PE) reads per lane (Illumina). A minimum of 30M PE150 reads were required per sample.

The FastQ files with paired-end reads were used as input for gene expression analysis on the LITOSeek platform (Novigenix SA). Of note, whole-blood RNA-seq data of patients in the discovery cohort have been previously published (20). Samples were re-sequenced and re-analyzed for this paper after optimization of the analyses pipeline.

### Data processing and quality check.

Sequence data quality was evaluated using FastQC (version 0.11.9; https://www.bioinformatics.babraham.ac.uk/projects/fastqc/) combined with MultiQC (version 1.11; https://seqera.io/multiqc/). Cutadapt (version 3.4; https://cutadapt.readthedocs.io/en/stable/index.html) was used to find and remove adapter sequences, primers, poly-A tails, and other types of unwanted sequence from high-throughput sequencing. Reads were aligned to the human genome assembly (GRCh38) along with its corresponding annotation from the Ensembl database (https://www.ensembl.org/index.html) using release 107. The pseudo-alignment and quantification of transcript abundance of the RNA-seq reads was done using Salmon (version 1.5.2; https://github.com/COMBINE-lab/salmon) with default parameters. All samples were used for downstream analysis.

### Data transformation and exploratory analysis.

Normalization for gene length, transcripts per million (TPM) values, was conducted as a step downstream in our analysis. Gene pseudo-counts from Salmon were imported into the R statistical computing environment (version 4.2.1; https://www.r-project.org) and subsequently filtered by excluding genes with less than 1 count per million (CPM) across all samples and with a coefficient of variance (CV) of 100, using the filtered.data function within the NOISeq R package (version 2.40.0). Following the initial gene data treatment, forward normalization was performed employing the variance-stabilizing transformation using the vst function, which is a feature of the DeSeq2 R package (version 1.36.0). Primary focus for exploratory data analysis centered on the vst-transformed values and the selected subset of genes from NOISeq. Principal component analysis (PCA) and scatter plots were applied to visualize the similarities and differences among samples.

### Differential gene expression and multivariate analysis.

Comprehensive DEA was performed using proprietary algorithms and curation of the DEGs. Three DEAs were performed: the first compared the OT samples of patients with CB with their paired BL samples, the second compared the OT and BL samples of patients with N-CB, and the third compared the OT samples of patients with CB versus patients with N-CB. Functional and network analyses of the DEGs were realized with STRING (version 12.0; https://string-db.org) and Cluster-Profiler (version 4.6.2; https://bioconductor.org/packages/release/bioc/html/clusterProfiler.html) to perform ORA, which allowed the identification of central biological pathways and biomarkers of response. STRING clusters were defined by MCL clustering (inflation parameter = 3) on the STRING online platform by inputting the longitudinal DEA CB DEGs. Significantly enriched ORA pathways were defined by an adjusted *P* value of 0.05 or less (Benjamini-Hochberg method). Additionally, the DEGs attributed to any enriched terms from the ORA results were extrapolated, enabling identification of the functionally relevant genes among all DEGs. Basic plots were performed with RStudio (version 4.2.1) and the correspondent R packages ggplot2 (version 3.5.0; https://ggplot2.tidyverse.org) and UpSetR (version1.4.0; https://cran.r-project.org/web/packages/UpSetR/index.html). Heatmaps were generated with ComplexHeatmap (version 2.14.0), ROCR (version 1.0-11) was used to plot ROC curves, and survminer (version 0.4.9) and survival (version 3.5-8) were used to generate Kaplan-Meier curves for PFS.

### Modeling.

To develop predictive models of CB to ICIs, the DEGs identified in the longitudinal CB DEA of the discovery cohort were used as input to generate multiple models, using several iterations of biomarker subset selection. Patients were distributed in discovery, test, and validation cohorts, based on the timing of enrollment and sample collection. The discovery cohort was used for biomarker discovery and model training, the test cohort for independent model testing and selection, and the validation cohort for final blinded validation of the model. To classify patients into predicted CB and predicted N-CB, we employed the sparse partial least squares method, which is particularly effective for small sample sizes and enhances model interpretability. The modeling process incorporated a resampling method, repeated cross-validation with 10 iterations, and a repeated *k*-fold cross-validation of 3 for the discovery dataset. Feature reduction was performed during each modeling iteration based on the initial feature list (DEGs) to identify the optimal model. This reduction was systematically applied by specifying feature selection within the ranges of 10, 15, and 20 features.

Each model’s performance was assessed by plotting the true positive rate against the false-positive rate at sensitivity and specificity thresholds of 90%. To classify samples as CB or N-CB, a 55% probability cutoff was used. The efficiency of the model was further verified by plotting Kaplan-Meier survival curves based on the model’s predictions, along with the corresponding hazard ratios and the distance between predictive curves of responders and nonresponders at 50% of the PFS. This process allowed identifying the best performing model (highest AUC and largest PFS separation) that comprised the 10-gene set. The identification of the 10 genes was therefore based on model performance upon feature selection from the DEGs identified in the longitudinal CB DEA.

### Multimodal modeling.

The multimodal model was optimized on the test cohort and blindly validated on the validation cohort. We did not use the discovery cohort (where the RNA model was trained) to avoid multimodal model overfitting. Specifically, for the development of a multimodal model based on RNA and ctDNA, RNA model prediction probabilities and ctDNA ratio values (ctDNA copies/mL at OT/ctDNA copies/mL at BL) were incorporated in the test cohort for threshold optimization. Specifically, ctDNA-based predictions were adjusted using the RNA model prediction if a patient’s ctDNA ratio value fell within an uncertainty range around the ctDNA ratio cutoff of 1. In such cases, the readout of the multimodal model for that specific patient sample would have been based on the RNA model. Vice versa, if the RNA model prediction probability was within an uncertainty range around the cutoff of 55%, the multimodal model readout would have been based on the ctDNA model. If both ctDNA ratio and prediction probability were falling into the respective uncertainty ranges, the RNA model would have been prioritized in the multimodal readout. Accordingly, multiple multimodal models were created by enlarging the uncertainty ranges for both ctDNA ratio and RNA model prediction probability. Each multimodal model performance was then assessed by calculating specificity and sensitivity, which was then compared to the standalone RNA model performance. The best combination of ctDNA ratio and predictive probability cutoffs defining the uncertainty ranges was selected in the test cohort, allowing for the highest increase in sensitivity and specificity compared to the standalone RNA model performance. Cutoffs were then applied to the validation cohort.

### Statistics.

Nonparametric data were analyzed by Wilcoxon’s test (paired or unpaired depending on the experimental setup). A *P* value of less than 0.05 was considered statistically significant. Differences in PFS in Kaplan-Meier curves were assessed by Mantel-Haenszel test. Each specific statistical test is reported for each experiment in the figure legends. Box-and-whisker plots are used to present the data, showing the median (line within box), 25th to 75th percentiles (box bounds), and variability outside the upper and lower quartiles (whiskers).

### Study approval.

The study was conducted in accordance with relevant guidelines and regulations, and approved by the CMO Radboudumc local medical ethics committee (local registration numbers 2016-3060 and 2020-6778). Written consent was obtained from all patients for the use of biomaterials. A flow diagram of the study is presented in Supplemental Figure 1A.

### Data availability.

Data displayed in the figures are available in the Supporting Data Values file. The processed ctDNA data are provided in Supplemental Dataset 1. High-throughput RNA-seq data set has been deposited under the following DOI: 10.5281/zenodo.14283210 (https://zenodo.org/records/14283210). The accessibility to the next-generation sequencing data generated from patient samples that support the findings of this study is restricted to protect human participant privacy and rights and preserve the scope of participants’ consent. Requests for data access should be addressed to the corresponding authors. All requests for raw and analyzed data will be promptly reviewed to verify whether the request is subject to any intellectual property, confidentiality obligations, or privacy’s restrictions to patient sensitive data. Any data and materials that can be shared will be released via a Data Transfer Agreement.

## Author contributions

SVW contributed to conceptualization, data curation, formal analysis, funding acquisition, validation, investigation, methodology, project administration, and writing the original draft of the manuscript. DC contributed to conceptualization, data curation, software, formal analysis, supervision, validation, investigation, visualization, methodology, project administration, and writing the original draft of the manuscript. SSFC contributed to data curation, software, formal analysis, validation, investigation, visualization, and methodology. IBAWTP and SHT contributed to data curation, formal analysis, validation, investigation, and methodology. JVI and LIK contributed to resources, investigation, and methodology. SP, SMB, and GC contributed to resources and data curation. NH contributed to software and methodology. IMO, TJS, TVV, MB, and MDF contributed to resources. MJLL contributed to supervision. SHE contributed to conceptualization, supervision, funding acquisition, investigation, and project administration. LC contributed to conceptualization, data curation, supervision, funding acquisition, validation, investigation, methodology, and project administration. PR contributed to conceptualization and supervision. NM contributed to conceptualization, resources, funding acquisition, validation, investigation, methodology, and project administration.

## Supplementary Material

Supplemental data

ICMJE disclosure forms

Supplemental data set 1

Supporting data values

## Figures and Tables

**Table 1:**
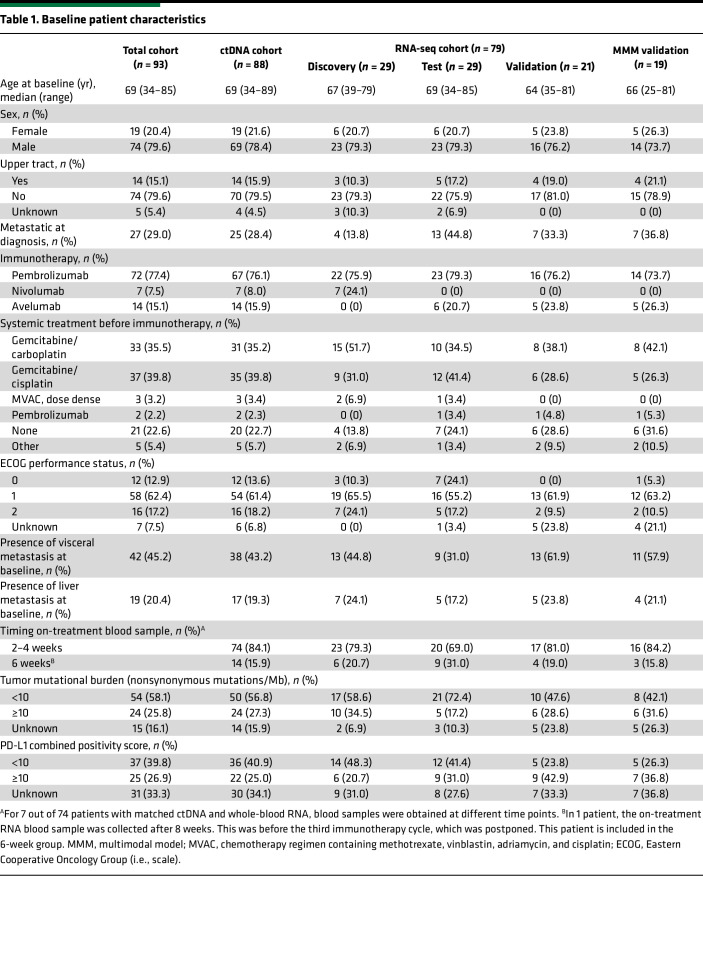
Baseline patient characteristics

## Version 1 01/30/2025

In-Press Preview

## Version 2 03/10/2025

Electronic publication

## References

[1] Bellmunt J (2017). Pembrolizumab as second-line therapy for advanced urothelial carcinoma. N Engl J Med.

[2] Powles T (2020). Avelumab maintenance therapy for advanced or metastatic urothelial carcinoma. N Engl J Med.

[3] Powles T (2024). ESMO Clinical Practice Guideline interim update on first-line therapy in advanced urothelial carcinoma. Ann Oncol.

[4] Powles T (2024). Enfortumab vedotin and pembrolizumab in untreated advanced urothelial cancer. N Engl J Med.

[5] van der Heijden MS (2023). Nivolumab plus gemcitabine–cisplatin in advanced urothelial carcinoma. N Engl J Med.

[6] Balar A V (2017). First-line pembrolizumab in cisplatin-ineligible patients with locally advanced and unresectable or metastatic urothelial cancer (KEYNOTE-052): a multicentre, single-arm, phase 2 study. Lancet Oncol.

[7] Balar AV (2023). Efficacy and safety of pembrolizumab in metastatic urothelial carcinoma: results from KEYNOTE-045 and KEYNOTE-052 after up to 5 years of follow-up. Ann Oncol.

[8] Powles T (2018). Atezolizumab versus chemotherapy in patients with platinum-treated locally advanced or metastatic urothelial carcinoma (IMvigor211): a multicentre, open-label, phase 3 randomised controlled trial. Lancet.

[9] Powles T (2019). Clinical efficacy and biomarker analysis of neoadjuvant atezolizumab in operable urothelial carcinoma in the ABACUS trial. Nat Med.

[10] Necchi A (2018). Pembrolizumab as neoadjuvant therapy before radical cystectomy in patients with muscle-invasive urothelial bladder carcinoma (PURE-01): an open-label, single-arm, phase II study. J Clin Oncol.

[11] Samstein RM (2019). Tumor mutational load predicts survival after immunotherapy across multiple cancer types. Nat Genet.

[12] Rui X (2019). Evaluation of PD-L1 biomarker for immune checkpoint inhibitor (PD-1/PD-L1 inhibitors) treatments for urothelial carcinoma patients: a meta-analysis. Int Immunopharmacol.

[13] Kilgour E (2020). Liquid biopsy-based biomarkers of treatment response and resistance. Cancer Cell.

[14] Powles T (2024). Pembrolizumab for advanced urothelial carcinoma: exploratory ctDNA biomarker analyses of the KEYNOTE-361 phase 3 trial. Nat Med.

[15] Tolmeijer SH (2023). Early on-treatment circulating tumor DNA measurements and response to immune checkpoint inhibitors in advanced urothelial cancer. Eur Urol Oncol.

[16] Herbst RS (2014). Predictive correlates of response to the anti-PD-L1 antibody MPDL3280A in cancer patients. Nature.

[17] Strickler JH (2021). Tumor mutational burden as a predictor of immunotherapy response: is more always better?. Clin Cancer Res.

[18] Fairfax BP (2020). Peripheral CD8^+^ T cell characteristics associated with durable responses to immune checkpoint blockade in patients with metastatic melanoma. Nat Med.

[19] Luoma AM (2022). Tissue-resident memory and circulating T cells are early responders to pre-surgical cancer immunotherapy. Cell.

[20] van Wilpe S (2021). Whole blood transcriptome profiling identifies DNA replication and cell cycle regulation as early marker of response to anti-PD-1 in patients with urothelial cancer. Cancers (Basel).

[21] Hendrickx W (2017). Identification of genetic determinants of breast cancer immune phenotypes by integrative genome-scale analysis. Oncoimmunology.

[22] Seitz RS (2022). Translation of the 27-gene immuno-oncology test (IO score) to predict outcomes in immune checkpoint inhibitor treated metastatic urothelial cancer patients. J Transl Med.

[23] Reijers ILM (2023). IFN-γ signature enables selection of neoadjuvant treatment in patients with stage III melanoma. J Exp Med.

[24] Argelaguet R (2018). Multi-omics factor analysis—a framework for unsupervised integration of multi-omics data sets. Mol Syst Biol.

[25] Sammut SJ (2022). Multi-omic machine learning predictor of breast cancer therapy response. Nature.

[26] Seymour L (2017). iRECIST: guidelines for response criteria for use in trials testing immunotherapeutics. Lancet Oncol.

[27] Raja R (2018). Early reduction in ctDNA predicts survival in patients with lung and bladder cancer treated with durvalumab. Clin Cancer Res.

[28] Goldberg SB (2018). Early assessment of lung cancer immunotherapy response via circulating tumor DNA. Clin Cancer Res.

[29] Herbreteau G (2020). Circulating tumor DNA as a prognostic determinant in small cell lung cancer patients receiving atezolizumab. J Clin Med.

[30] Váraljai R (2019). Application of circulating cell-free tumor DNA profiles for therapeutic monitoring and outcome prediction in genetically heterogeneous metastatic melanoma. JCO Precis Oncol.

[31] Anagnostou V (2019). Dynamics of tumor and immune responses during immune checkpoint blockade in non–small cell lung cancer. Cancer Res.

[32] Kansara M (2023). Early circulating tumor DNA dynamics as a pan-tumor biomarker for long-term clinical outcome in patients treated with durvalumab and tremelimumab. Mol Oncol.

[33] Richard C (2024). Abstract 7611: Circulating blood RNAseq detects activation of immune system and predicts response to immunotherapy in bladder cancer. Cancer Res.

[34] Huang AC (2017). T-cell invigoration to tumour burden ratio associated with anti-PD-1 response. Nature.

[35] Galsky MD (2024). Characterization of complete responders to nivolumab + gemcitabine-cisplatin vs gemcitabine-cisplatin alone and patients with lymph node–only metastatic urothelial carcinoma from the CheckMate 901 trial. J Clin Oncol.

[36] Schwartz LH (2016). RECIST 1.1–Update and clarification: from the RECIST committee. Eur J Cancer.

[37] Agarwal N (2014). Six-month progression-free survival as the primary endpoint to evaluate the activity of new agents as second-line therapy for advanced urothelial carcinoma. Clin Genitourin Cancer.

[38] Annala M (2018). Circulating tumor DNA genomics correlate with resistance to abiraterone and enzalutamide in prostate cancer. Cancer Discov.

