# Supplementary material for: Multimodal integration of blood RNA and ctDNA reflects response to immunotherapy in metastatic urothelial cancer
Source: JCI Insight. 2025 Jan 30;10(5):e186062. doi: 10.1172/jci.insight.186062 (PMC11949011; doi:10.1172/jci.insight.186062)
Supplement: Supplemental data [file jciinsight-10-186062-s218.pdf]

Figure S1

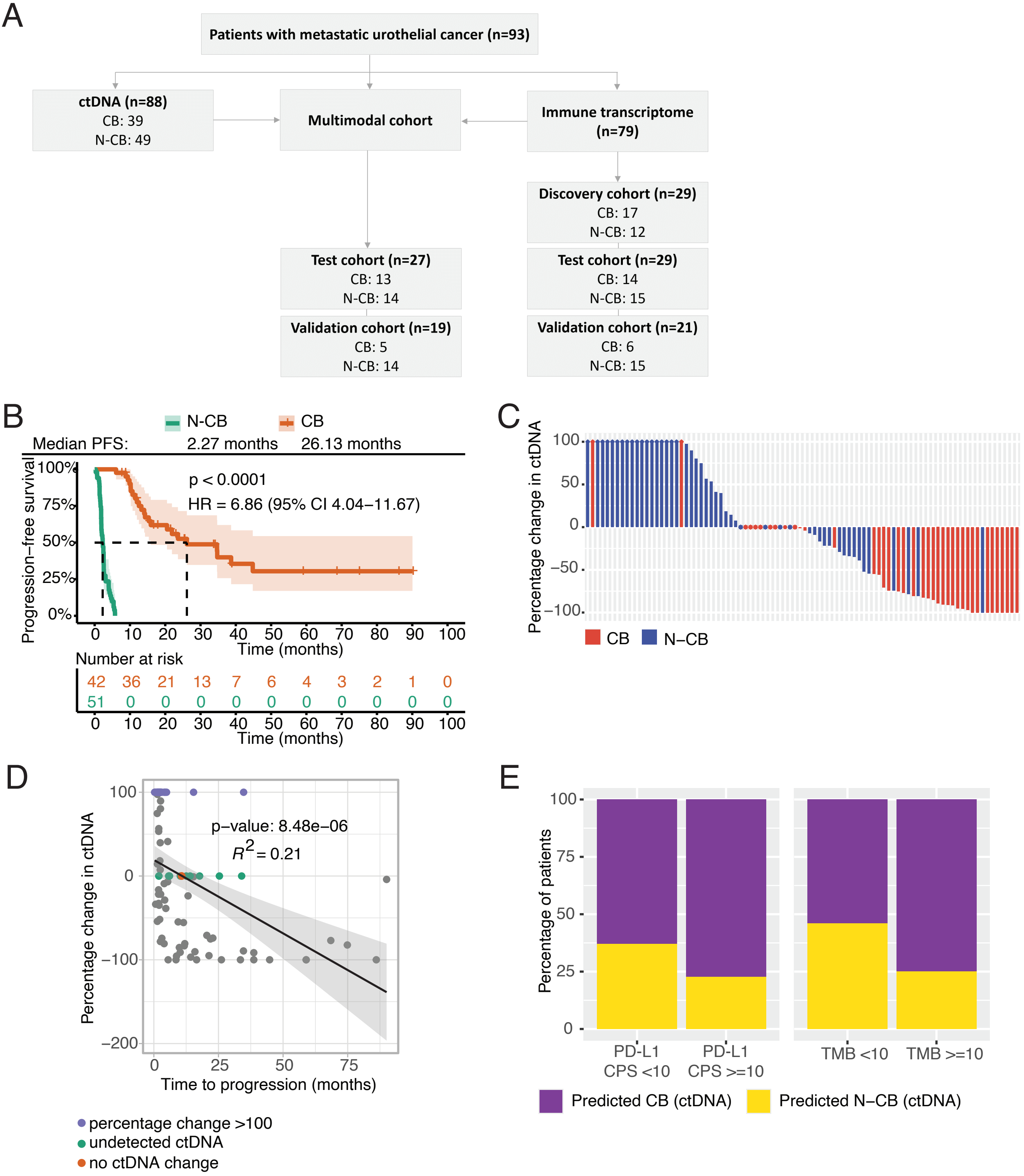
A
Patients with metastatic urothelial cancer (n=93)
ctDNA (n=88)
CB: 39
N-CB: 49
Multimodal cohort
Immune transcriptome (n=79)
Discovery cohort (n=29)
CB: 17
N-CB: 12
Test cohort (n=27)
CB: 13
N-CB: 14
Test cohort (n=29)
CB: 14
N-CB: 15
Validation cohort (n=19)
CB: 5
N-CB: 14
Validation cohort (n=21)
CB: 6
N-CB: 15
B
N-CB
CB
Median PFS:
2.27 months
26.13 months
p < 0.0001
HR = 6.86 (95% CI 4.04–11.67)
Progression−free survival
100%
75%
50%
25%
0%
0 10 20 30 40 50 60 70 80 90 100
Time (months)
Number at risk
42 36 21 13 7 6 4 3 2 1 0
51 0 0 0 0 0 0 0 0 0 0
0 10 20 30 40 50 60 70 80 90 100
Time (months)
C
Percentage change in ctDNA
100
50
0
−50
−100
CB
N−CB
D
Percentage change in ctDNA
100
0
−100
−200
p−value: 8.48e−06
$R^2 = 0.21$
0 25 50 75
Time to progression (months)
percentage change >100
undetected ctDNA
no ctDNA change
E
Percentage of patients
100
75
50
25
0
PD-L1 CPS <10
PD-L1 CPS >=10
TMB <10
TMB >=10
Predicted CB (ctDNA)
Predicted N−CB (ctDNA)

Figure S2

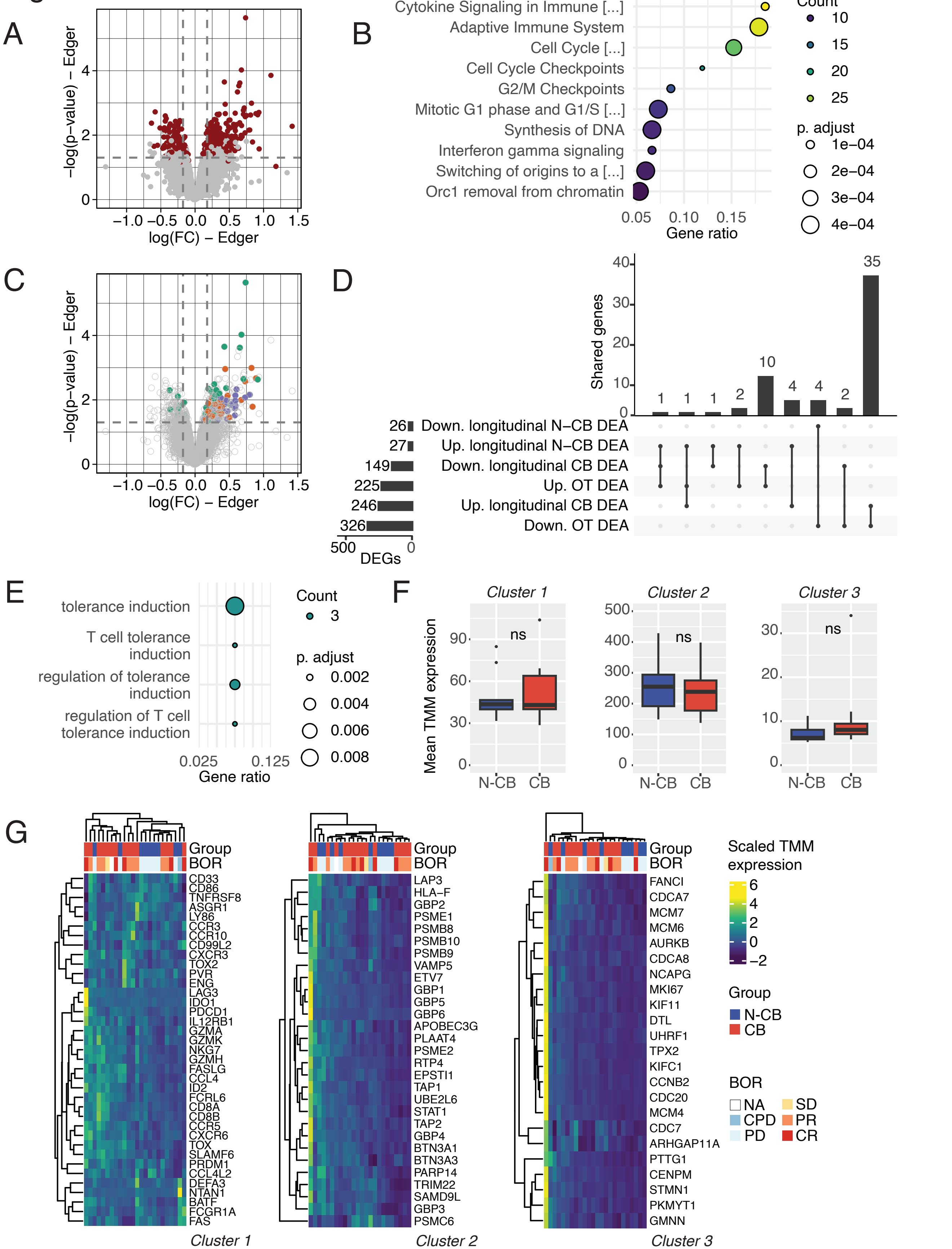
A
−log(p-value) − Edger
log(FC) − Edger
B
Cytokine Signaling in Immune [...]
Adaptive Immune System
Cell Cycle [...]
Cell Cycle Checkpoints
G2/M Checkpoints
Mitotic G1 phase and G1/S [...]
Synthesis of DNA
Interferon gamma signaling
Switching of origins to a [...]
Orc1 removal from chromatin
Gene ratio
Count
10
15
20
25
p. adjust
1e−04
2e−04
3e−04
4e−04
C
−log(p-value) − Edger
log(FC) − Edger
D
Shared genes
Down. longitudinal N−CB DEA
Up. longitudinal N−CB DEA
Down. longitudinal CB DEA
Up. OT DEA
Up. longitudinal CB DEA
Down. OT DEA
DEGs
E
tolerance induction
T cell tolerance induction
regulation of tolerance induction
regulation of T cell tolerance induction
Gene ratio
Count
p. adjust
F
Cluster 1
Cluster 2
Cluster 3
Mean TMM expression
ns
N-CB
CB
G
Group
BOR
Cluster 1
Cluster 2
Cluster 3
Scaled TMM expression
Group
N-CB
CB
BOR
NA
CPD
PD
SD
PR
CR

Figure S3

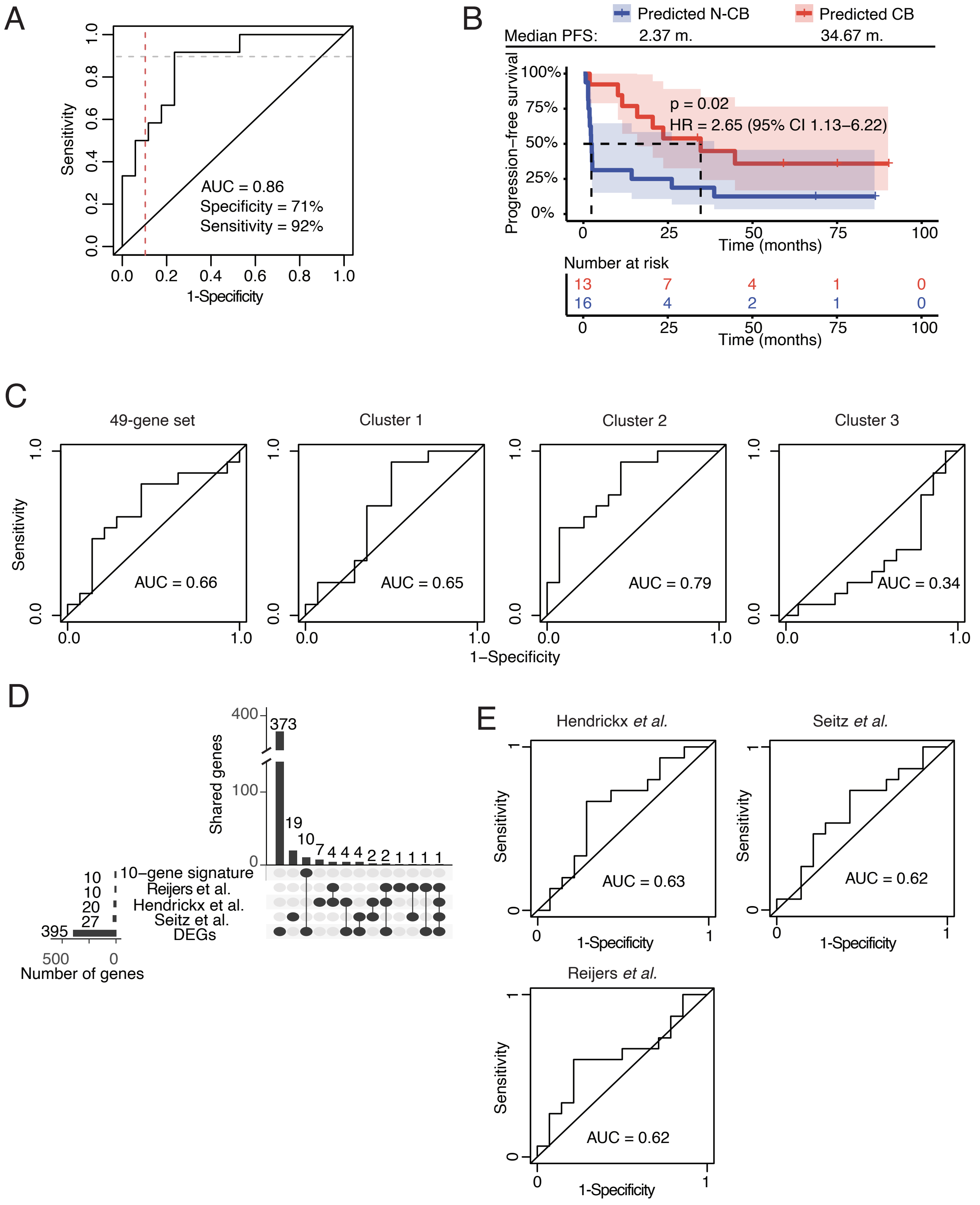
A
Sensitivity
1-Specificity
AUC = 0.86
Specificity = 71%
Sensitivity = 92%
B
Predicted N-CB
Predicted CB
Median PFS: 2.37 m. 34.67 m.
Progression−free survival
p = 0.02
HR = 2.65 (95% CI 1.13–6.22)
Time (months)
Number at risk
13 7 4 1 0
16 4 2 1 0
C
49-gene set
Cluster 1
Cluster 2
Cluster 3
AUC = 0.66
AUC = 0.65
AUC = 0.79
AUC = 0.34
1−Specificity
D
Shared genes
373
19
10
7 4 4 4 2 2 1 1 1 1
10–gene signature
Reijers et al.
Hendrickx et al.
Seitz et al.
DEGs
10
10
20
27
395
Number of genes
E
Hendrickx et al.
Seitz et al.
Reijers et al.
AUC = 0.63
AUC = 0.62
AUC = 0.62

Figure S4

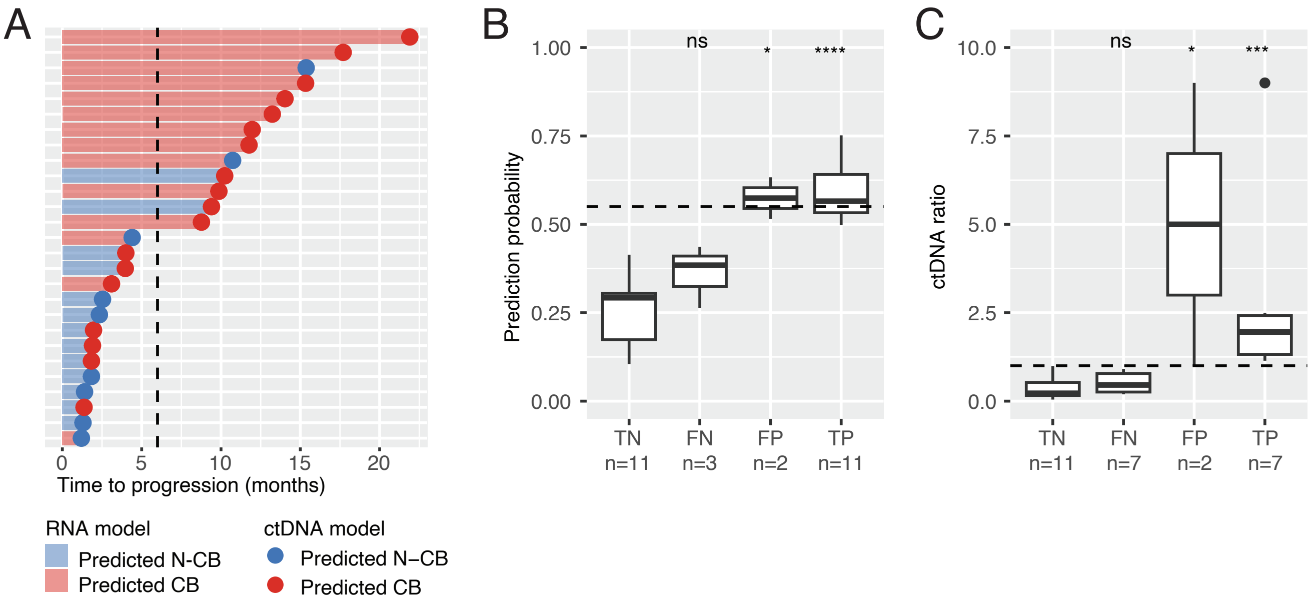
A
B
C
0
5
10
15
20
Time to progression (months)
RNA model
Predicted N-CB
Predicted CB
ctDNA model
Predicted N–CB
Predicted CB
Prediction probability
1.00
0.75
0.50
0.25
0.00
ns
*
****
TN
n=11
FN
n=3
FP
n=2
TP
n=11
ctDNA ratio
10.0
7.5
5.0
2.5
0.0
ns
*
***
TN
n=11
FN
n=7
FP
n=2
TP
n=7
